# New Paper Spinal Brace

**Published:** 1879-10

**Authors:** Morgan Vance


### NEW PAPER SPINAL BRACE.

In the New York County Medical Society, Dr. Morgan Vance showed recently a spinal brace, made of paper, glue and steel springs, which he considered more advantageous than the plaster of Paris bandages.

An ordinary plaster jacket is first made, allowed to harden, and then cut down, the inner surface carefully smoothed, and then a solid cast made by filling the jacket with plaster of Paris and mortar.

Over this cast the paper brace is made in the following manner: Canton flannel, intended to form the inner surface of the brace, is stretched tightly over the cast, and to its outer surface a paste is applied, consisting of one part of white glue, two parts of oxide of zinc, and six parts of hot water. Over this there is put horizontal strips of brown manilla paper, one and a half inches wide, and long enough to reach a little more than half way around the cast, each strip overlapping the other, until the cast has been covered in front and behind. Over this steel springs, such as are used in hoop-skirts, are placed one and a half inches apart, held in place by stout thread, then another vertical layer of paper, and finally a roller tightly applied.

This requires twenty-four to forty-eight hours to harden. After this it is cut down in front, lined, bound with thin leather along the edges, and eyelets inserted down the front. For the purpose of ventilation, pieces about the size of a dollar are cut out, care being taken to avoid the springs. The advantages of this brace are: 1, lightness; 2, ventilation; 3, durability; 4, perfect fit; 5, that it can be easily removed and replaced again; 6, that we by insertion of a rubber band may expect continuous pressure over the projecting side of the thorax in lateral curvature.—*Hospital Gazette*.

